# Meetings of Societies

**Published:** 1934

**Authors:** 


### Meetings of Societies

### Bristol Medico-Chirurgical Society

THE sixth meeting of the session was held in the University on Wednesday, 14th March, the President, Dr. H. Elwin Harris, in the Chair. Forty members were present.

Dr. F. J. A. Mayes showed skiagrams, Dr. A. D. Fraser pathological specimens, and Mr. E. Watson-Williams a case of **Keratosis Pharyngis.**

Dr. H. Elwin Harris (Jun.) read a short communication on a case of **Double Empyæma,** which was discussed by the President and by Mr. W. A. Jackman.

Mr. A. J. M. Wright then read a paper on "**Focal Infection,**" published on p. 109 of this issue. The paper was discussed by the President, Dr. P. Watson-Williams, Dr. Rayner, Dr. A. T. Todd, and Dr. J. I. Noble.

---

The seventh meeting of the session was held in the University on Wednesday, 11th April, the President in the Chair. Forty-six members were present.

Mr. F. G. Bergin showed skiagrams and Dr. A. L. Taylor pathological specimens.

Mr. A. W. Adams showed a patient on whom he had operated three years previously for **Sarcoma of the Femur,** and discussed the differential diagnosis; his remarks are published on p. 117.

Professor R. J. Brocklehurst read a communication on the subject of "**The Expulsion of Bile,**" published on p. 131 of this issue. The paper was discussed by the President and Mr. C. A. Moore.

---

The eighth and last meeting of the session was held in the University on Wednesday, 9th May, the President in the Chair.

Dr. A. D. Fraser showed pathological specimens and Mr. E. Watson-Williams (*a*) a very large foreign body removed from the gullet, (*b*) a case of **Lymphocytoma Malignum** affecting the tonsil, and discussed the varieties of sarcoma that might be seen in the tonsils.

Dr. R. C. Clarke read a short paper on "**Pink Disease.**"

Dr. G. B. Bush read a paper on "**The Clinical Importance of the Intervertebral Discs.**" This was discussed by the President, Mr. F. G. Bergin, Mr. K. H. Pridie, Mr. D. Wood, Mr. A. R. Short, Dr. R. M. Forrester-Brown and Dr. R. C. Clarke.

